# Patient-Specific Metrics of Invasiveness Reveal Significant Prognostic Benefit of Resection in a Predictable Subset of Gliomas

**DOI:** 10.1371/journal.pone.0099057

**Published:** 2014-10-28

**Authors:** Anne L. Baldock, Sunyoung Ahn, Russell Rockne, Sandra Johnston, Maxwell Neal, David Corwin, Kamala Clark-Swanson, Greg Sterin, Andrew D. Trister, Hani Malone, Victoria Ebiana, Adam M. Sonabend, Maciej Mrugala, Jason K. Rockhill, Daniel L. Silbergeld, Albert Lai, Timothy Cloughesy, Guy M. McKhann, Jeffrey N. Bruce, Robert C. Rostomily, Peter Canoll, Kristin R. Swanson

**Affiliations:** 1 Department of Neurological Surgery, Northwestern University Feinberg School of Medicine, Chicago, Illinois, United States of America; 2 Department of Pathology, University of Washington, Seattle, Washington, United States of America; 3 Radiation Oncology, University of Washington, Seattle, Washington, United States of America; 4 Department of Neurosurgery, Columbia University, New York, New York, United States of America; 5 Department of Neurology, University of Washington, Seattle, Washington, United States of America; 6 Department of Neurological Surgery, University of Washington, Seattle, Washington, United States of America; 7 Department of Neurology, University of California Los Angeles, Los Angeles, California, United States of America; 8 Department of Pathology and Cell Biology, Columbia University, New York, New York, United States of America; 9 Nancy and Buster Alvord Brain Tumor Center, University of Washington, Seattle, Washington, United States of America; 10 Northwestern Brain Tumor Institute, Robert H. Lurie Comprehensive Cancer Center, Chicago, Illinois, United States of America; University of Michigan School of Medicine, United States of America

## Abstract

**Object:**

Malignant gliomas are incurable, primary brain neoplasms noted for their potential to extensively invade brain parenchyma. Current methods of clinical imaging do not elucidate the full extent of brain invasion, making it difficult to predict which, if any, patients are likely to benefit from gross total resection. Our goal was to apply a mathematical modeling approach to estimate the overall tumor invasiveness on a patient-by-patient basis and determine whether gross total resection would improve survival in patients with relatively less invasive gliomas.

**Methods:**

In 243 patients presenting with contrast-enhancing gliomas, estimates of the relative invasiveness of each patient's tumor, in terms of the ratio of net proliferation rate of the glioma cells to their net dispersal rate, were derived by applying a patient-specific mathematical model to routine pretreatment MR imaging. The effect of varying degrees of extent of resection on overall survival was assessed for cohorts of patients grouped by tumor invasiveness.

**Results:**

We demonstrate that patients with more diffuse tumors showed no survival benefit (P = 0.532) from gross total resection over subtotal/biopsy, while those with nodular (less diffuse) tumors showed a significant benefit (P = 0.00142) with a striking median survival benefit of over eight months compared to sub-totally resected tumors in the same cohort (an 80% improvement in survival time for GTR only seen for nodular tumors).

**Conclusions:**

These results suggest that our patient-specific, model-based estimates of tumor invasiveness have clinical utility in surgical decision making. Quantification of relative invasiveness assessed from routinely obtained pre-operative imaging provides a practical predictor of the benefit of gross total resection.

## Introduction

High grade gliomas are diffusely invasive primary brain tumors known for their resistance to therapeutic intervention. The benefit of extensive resection of invasive gliomas has long been debated (c.f, [1]–[4]) and the diffuse nature of the disease precludes a surgical cure, as even hemispherectomies are followed by tumor recurrence [5], [6]. Several studies have identified factors correlated with post-operative survival, such as tumor location and volume, measures of the patient's clinical status such as the Karnofsky Performance Score (KPS), and patient age [1], [7]–[9]. Given the significant heterogeneity in tumor growth and response to treatment among individual patients, a large patient sample is essential when assessing the variable benefit of cytoreductive surgery. Although there is some conflicting data in the literature, recent population level studies of at least 400 patients each have shown evidence for incremental survival benefits with a larger extent of resection (EOR) [1], [2], [4], [10]. The comprehensive nature and large scope of these retrospective studies provides insight into the differential benefits of resection at a population level. Here we expand on this work by retrospectively quantifying the degree of diffuse invasion across patients and determining whether this metric can discriminate those patients that benefited more from extensive surgical resection. We hypothesized that the patients with the less invasive tumor proliferation will demonstrate a relatively larger benefit from gross total resection (GTR).

Recent studies supporting the clinical importance of resection have been accompanied by technological advances that improve neurosurgeons' ability to safely remove the maximum amount of tumor. Intraoperative MRI guidance, ^11^C-methionine PET imaging, and 5-aminolevulinic acid-induced fluorescence are among the tools currently used to optimize glioma resection [11]–[14]. Determining the survival benefit that results from increasing the EOR on a case-by-case basis could help direct the use of these advanced surgical adjuncts toward those patients who stand to gain the most from their application and, importantly, identify patients that are unlikely to benefit.

**Figure 1:**
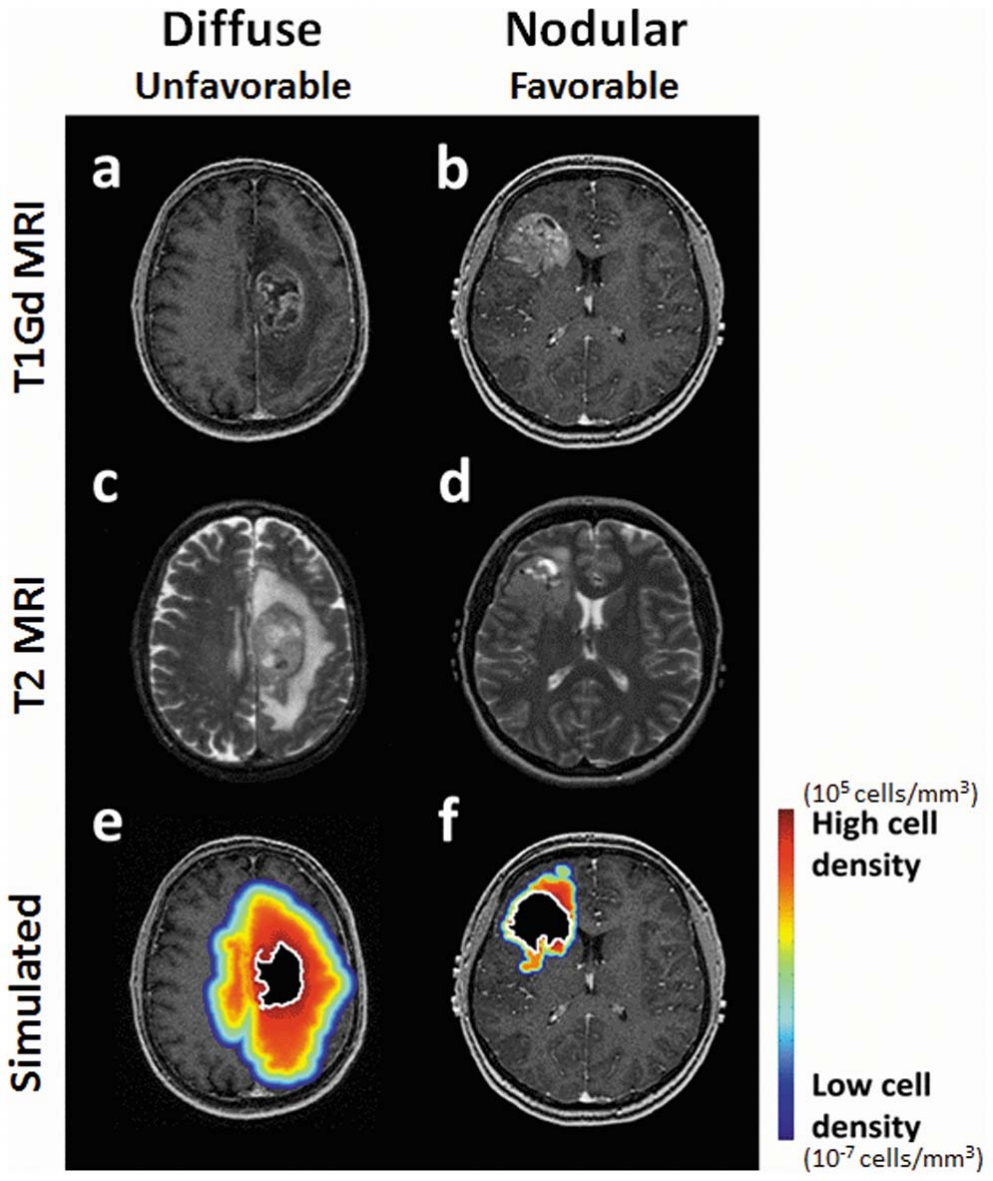
Patient-Specific Simulations of Tumor Cell Distribution and Density for both a Relatively Diffuse and a Relatively Nodular Glioblastoma. T1Gd and T2 MRIs for two newly diagnosed glioblastoma patients, one relatively diffuse with a low ρ/D (a,c) and one more nodular with a high ρ/D (b,d). A simulation of the diffuse glioma extent predicted by the patient-specific simulation for the diffuse (low ρ/D) patient (e) and the more nodular (high ρ/D) patient (f) is overlayed on the T1Gd MRI with red and blue indicating high and low (but nonzero) glioma cell density, respectively. The effect of GTR is shown as a black region with a white outline and highlights the significant diffuse extent of glioma cells remaining post-GTR. In the more nodular (high ρ/D) case, GTR removes 75% of the pre-treatment glioma cells leaving 8.4e8 cells while in the diffusely invasive (low ρ/D) case, GTR removes only 27% of the pre-treatment glioma cells leaving 4.2e9 cells, an order of magnitude higher than the nodular case. The large number of tumor cells remaining after resection of a diffuse tumor drives recurrence.

Glioma patients are routinely evaluated with MR imaging which only captures the “tip of the iceberg” of the tumor's diffusion profile. Specifically, gadolinium enhanced T1-weighted (T1Gd) MR imaging outlines the leaky angiogenic neovasculature associated with the highly cellular regions of glioma and T2-weighted (T2) MRI reveals diffusely invaded glioma cells and associated edema. Neither imaging technique can reveal the full extent of the glioma invasion. Coincident with the evolving literature on the potential benefits of extensive resection, we, and other researchers at the interface of the quantitative and clinical sciences, have been developing patient-specific biomathematical modeling of glioma proliferation and invasion. Specifically, by incorporating patient-specific mathematical modeling and data from routine MR imaging, we have developed techniques for determining the extent of diffuse invasion invisible on routine MRI in individual patients [15]–[22]. Over the last decade, we have applied these techniques extensively to quantify patient-specific metrics of net proliferation rate (ρ) and net invasion rate (D) which inform patient-specific simulations of glioma growth and progression within the human brain (c.f., Figure 1). Although these simulated glioma cell density distributions are estimated by simplifying assumptions regarding the role of T1Gd and T2 MRI in detecting different glioma cell densities, these methods have been highly successful in generating clinically relevant patient-specific metrics of tumor aggressiveness [19]–[21]. Postmortem verification of model predictions demonstrate that cell density simulations recapitulate the histologically-observed diffuse invasion of glioma cells peripheral to the imaging abnormality [23], Furthermore, the patient-specific model predictions are prognostically significant [18], [20]. The patient-specific metrics of net proliferation rate, ρ, and net invasion rate D have been quantitatively correlated to other measures of biological aggressiveness including hypoxic tumor burden assessed on [18F] fluoro-misonidazole (FMISO) PET [21] allowing predictive simulation of patient-specific FMISO-PET images strikingly similar to the actual clinical observations [24]. In a study extending the modeling approach to incorporate radiotherapy [20], the model has been shown to predict patient-specific radiosensitivity using routine pre-treatment clinical imaging information [22]. In a study of 70 patients treated with various extents of resection, our modeling approach accurately predicted the *population-level* survival difference between the biopsy/subtotal resection (Bx/STR) populations and the gross-total resection population [19] but this study was insufficiently powered to allow for cohorting patients according to their relative invasiveness (ρ/D) to assess the benefit of more extensive resection.

In the current study, we applied our novel mathematical modeling approach to a cohort of 252 patients with contrast-enhancing tumor on pre-treatment MRI. We studied the relationship between patients' relative tumor invasiveness and the benefit of GTR, defined as complete removal of the abnormality visible on T1Gd. To demonstrate the utility of our approach in clinical decision-making, we included contrast-enhancing gliomas of all grades (at presentation) to reflect the perspective of the neurosurgeon, who must make surgical decisions prior to histological assessment of tumor grade. Further, although previous population-level studies of resection have set benchmarks for the EOR (measured in terms of percent of the gadolinium-enhanced T1-weighted, T1Gd, MRI abnormality removed) needed to predict a positive post-operative prognosis, these scales do not account for patient-specific tumor growth rates or invasiveness [1], [9].The relationship between the T2∶T1Gd ratio (to infer information regarding the relative invasiveness of the disease) and survival has been investigated by others [8], but our work advances this type of analysis by calculating a patient-specific measure of the diffusion and proliferation rates, which we used to estimate the number of glioma cells remaining after surgery. Our hypothesis is that this transition from population-level studies of surgical benefit to patient-specific analysis yields information that can be used clinically to determine the benefit a particular patient will receive from more extensive resection. To our knowledge, this is the first application of patient-specific mathematical modeling in neuro-oncology to study such a large (n>200) cohort of patients leading to results that have significant implications for the routine clinical management of contrast-enhancing gliomas.

## Methods

### Patients and Clinical Data

We consider a cohort of 252 contrast-enhancing glioma patients at first diagnosis in our observational study, approved by the University of Washington institutional review board. Written consent was obtained and securely stored for the duration of the study per the principles of the Declaration of Helsinki. Pathological grade information at diagnosis was available for 247 patients out of the 252 (220 grade IV, 21 grade III, six grade II and five patients of unknown grade). Of the 252 patients, 211 were diagnosed, treated or consented at the University of Washington Medical Center, 35 at Columbia University, and six at University of California at Los Angeles Medical Center. Nine patients were excluded due to unconfirmed extent of resection, due to lack of post-operative imaging and imaging report. All of these patients had a pre-treatment T1Gd and T2/FLAIR image from the same time point. Extent of resection was classified as GTR, STR or Bx based on post-operative radiology report (N = 243). In addition, we quantified the extent of resection as the percent of T1Gd resected using pre- and post-operative imaging, when available (N = 181).

### Model Parameter Calculation

Our analysis employs a mathematical model that equates rate of change of glioma cell concentration to net invasion and net proliferation (a classical conservation-diffusion equation) [25], [26].
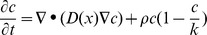
where

c = tumor or cell density at location x and time t
D = the dispersal of tumor cells
ρ = net proliferation rate of tumor cells
k = cell carrying capacity
x = location in the brain

**Figure 2:**
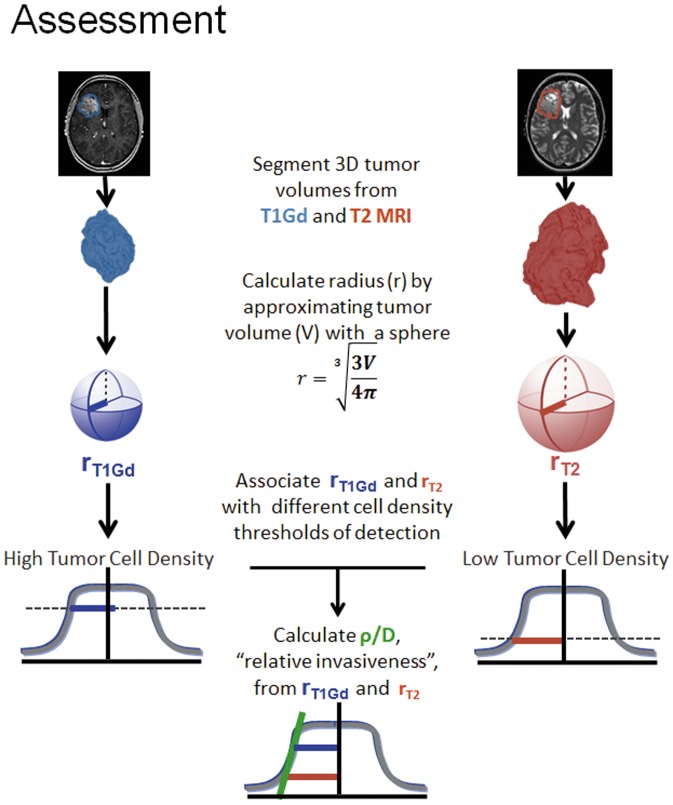
ρ/D Assessment. This figure presents an overview of how the “relative invasiveness,” or ρ/D, is obtained. Tumor volumes are segmented from T1Gd and T2 MRI. The measured volume is approximated with a sphere in order to obtain a radius. The T1Gd and T2 radii are associated with different levels of detection, with T2 at low tumor cell density and T1Gd abnormality associated with high tumor cell density. The relationship between these two radii describes the steepness of the tumor cell profile, or “relative invasiveness.”

**Figure 3:**
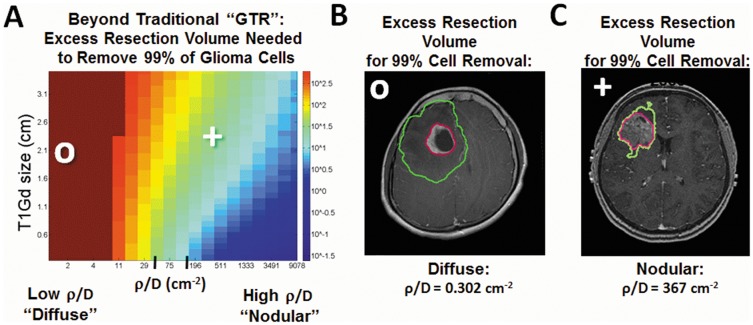
Extent of resection necessary to attain significant reduction in glioma cell burden. A) Simulated predictions of the additional resection volume beyond GTR of the T1Gd abnormality needed to achieve 99% reduction in tumor cell burden, a 2 log kill of tumor cells. The color scale maps to the additional volume (ranging from 10^−1.5^ to 10^2.8^, corresponding to hemispherectomy of an approximately 1200 cm^3^ brain volume) outside of the T1Gd abnormality needed to attain desired tumor cell removal. The cutoffs between low and moderate ρ/D (37.2 cm^−2^) and moderate and high ρ/D (135 cm^−2^) are displayed as tick marks on the horizontal axis. These theoretical resection margins necessary to achieve a 2 log kill of tumor cells are visualized for b) a diffuse tumor which requires an extra 237 cc of tissue (green contour) to be removed beyond a conventional GTR of the T1Gd abnormality (pink contour). In contrast, only 25 cc of brain tissue needs to be resected to remove 99% of the glioma cells in the more nodular glioma (c).

This model portrays the leading edge of the glioma as an advancing traveling wave. The shape and the speed of this wavefront are individualized to each patient by estimating the net rates of diffusion (D) and proliferation (ρ) prior to treatment byy associating the T1Gd and T2 enhancing regions as corresponding to different levels of cell density on this wave. Given sizes on T1Gd and T2 MRI one can calculate a measure of invasiveness, ρ/D, which is related to the ‘gradient’ between these two assumed cell density levels (Figure 2). This relationship is known to be highly non-linear and provides a unique individual profile of diffuse invasion at the leading edge of the imageable tumor [23] [15]–[17], [20], [27], [28], [15]–[17], [20]–[22], [27] which can be used to generate patient-specific invasion profiles from pre-treatment MRIs (high ρ/D: Figure 1a,c,e. low ρ/D: Figure 1b,d,f). The most diffuse tumors (low ρ/D) are those with a low proliferative potential (ρ) relative to their invasive potential (D); the least diffuse, most nodular, tumors (high ρ/D) are those with a high proliferative potential relative to their invasive potential. We estimated tumor volumes from T1Gd and T2/FLAIR MRIs using a semi-automated image segmentation method we developed previously in Matlab [22]. The time between images used for analyzing resection and tissue diagnosis was minimized in order to capture tumor dynamics just prior to therapeutic intervention. The mean time difference was 1.7 days, and the median was 4.5 days (0–112 day range). Using the pre-treatment MRIs and the invasiveness metric, we can simulate the tumor cell density over space even peripheral to the abnormality seen on imaging (heat map in Figure 1), and estimate the resection margin needed to remove 99% of the glioma (green contours in Figure 3b and 3c). Specifically, by computationally masking out the region of the T1Gd abnormality to approximate a gross total resection (Figures 1e and 1f), we are able to visualize and quantify the predicted extent of glioma cell distribution following removing varying extents of resection – [29]. This reveals a drastically different pattern of residual disease for the more diffuse glioma (low ρ/D – Fig. 1e) when compared to the more nodular glioma (high ρ/D – Figure 1f). Using measurements of the tumor volume resected, number of residual cells left was estimated for patients with post-operative imaging. Within each invasiveness cohort, each possible cutoff for number of cells remaining was used to separate patients into groups with relatively large and small estimated numbers of residual tumor cells. Log rank tests assessed the difference between survival curves for these groups within each cohort, for each cutoff.

### Statistical Analysis

We used the PASW Statistics 18 software package to obtain descriptive statistics. The Kaplan-Meier survival analysis (log-rank test) was performed in Matlab. For all statistical tests we considered P-values less than or equal to 0.05 to be significant.

## Results

We performed a survival analysis to compare GTR to STR/Bx in the overall patient cohort, as confirmed by post-operative imaging, revealing an improvement in overall survival approaching statistical significance amongst those receiving GTR (p = 0.08). To test the hypothesis that a subset of patients drive the observed survival benefit of GTR, patients were cohorted according to relative invasiveness. All 243 contrast-enhancing glioma patients were equally divided into three cohorts according to the relative level of invasiveness of their disease: “diffuse” with a low proliferative to invasive ratio (low ρ/D, 0.07–0.38 mm^−2^), moderately invasive with a moderate proliferative to invasive ratio (mid ρ/D, 0.38–1.30 mm^−2^) and “nodular” with a high proliferative to the invasive ratio (high ρ/D,1.30–294.1 mm^−2^). Dividing the population in this way provides cohorts with sufficient N to allow for survival analysis across scales of relative invasiveness whereas progression-free survival data, available in 102 patients but failed to reach significance in either the overall or sub cohorts.

Age, KPS, grade and treatment data are summarized in Table 1. There was no statistical difference in age across invasiveness cohorts (P = 0.93, ANOVA test). There was no difference in KPS between invasiveness cohorts (P = 0.25, ANOVA test). The mean KPS scores at diagnosis in the GTR and Bx/STR groups were 83.7 and 78.1 respectively, showing a slight reflection of the bias toward higher KPS expected in patients who are candidates for GTR. There was no statistical difference in proportion of patients receiving GTR versus Bx/STR between cohorts (P = 0.39, Proportion chi-square test).There was no statistical difference in radiation dose received across cohorts (P = 0.22, ANOVA). Post-operative complications were rare with 1 confirmed in each invasiveness cohort with two cranial infections and one fever of unknown origin (presumed toxic encephalopathy). The median radiation dose in all three groups was 6000 cGy, with means of 5827 cGy, 5624 cGy, and 6004 cGy for the diffuse, moderate, and nodular groups respectively. There was no statistical difference in proportion of patients who received concurrent Temozolomide with radiation or steroid use between patients who had a GTR versus patients who had less extensive resections (P = 0.73 and P = 0.62 respectively, Proportion chi-square test).

**Table 1:** Clinical Data Table.

		Diffuse (n = 81)		Moderate (n = 80)		Nodular (n = 82)	
		Bx/STR*: N = 53(65%)	GTR*: N = 28 (35%)	Bx/STR*: N = 51(64%)	GTR*: N = 29(36%)	Bx/STR*: N = 45(55%)	GTR*: N = 37(45%)
Age**	Median (Range)	55 (24, 88)	58 (34, 86)	58 (22, 81)	58 (22, 83)	58 (83, 19)	58 (20, 76)
Sex	N (F, M)	53 (15, 38)	28 (8, 20)	51 (18, 33)	29 (15, 14)	45 (24, 21)	37 (13, 24)
Race	N (unknown, C, A, H, B)	53 (22, 28, 1, 2, 0)	28 (11, 15, 1, 1, 0)	51 (29, 21, 1, 0, 0)	29 (9, 19, 1, 0, 0)	45 (28, 15, 2, 0, 0)	37 (12, 22, 1, 1, 1)
KPS**	Median (Range) [N]	80 (30, 100) [37]	80 (50, 100) [21]	80 (60, 100) [43]	80 (50, 100) [23]	80 (50, 100) [38]	90 (70, 100) [32]
XRT Dose**	Median (Range) [N]	6000 (3000, 6400) [39]	6000 (1800, 6120) [18]	5940 (180, 6300) [35]	6000 (1440, 6400) [21]	6000 (3900, 6900) [40]	6000 (5940, 6480) [29]
Dx in 90s	N	12	10	11	9	14	7
Dx in 2000s	N	40	17	39	19	29	27
Pre-op Steroid*	N	22	10	14	11	21	11
Concurrent TMZ*?	N	22	5	14	8	15	13

Median age and range, distribution of males and females, race (unknown, Caucasian, Asian, Hispanic, Black), median KPS and range, median XRT Dose and range, number of patients diagnosed in the 90's, number of patients diagnosed in 2000 or later, number receiving preoperative steroids, and number of patients who received concurrent Temozolomide with XRT are shown. Proportion chi-square tests were performed to compare steroid administration, concurrent TMZ, and proportion of patients who received GTR vs. STR/Bx between the three invasiveness cohorts. No statistical difference at the P = 0.05 significance level was found in any of these variables between cohorts. ANOVA tests were performed to compare KPS scores, age, and XRT doses between invasiveness cohorts. No difference at the P = 0.05 significance level was found in any of these variables between cohorts.

* - Indicates no significant difference (p≤0.05) between cohorts in this variable, per Proportion Chi-square test.

** - Indicates no significant difference (p≤0.05) between cohorts in this variable, per ANOVA test.

Table 2 shows the multivariate and univariate Cox proportion-hazards regression analysis. Additional Cox proportion-hazards regression analysis performed for each invasiveness cohort is detailed in Table S1. Age, KPS, XRT dose, concurrent Temozolomide, tumor grade, race, EOR, decade of diagnosis, and T1Gd lesion size have a significant effect on survival at the P = 0.05 level of significance. Even given the challenge of multivariate analysis on a relatively modest patient cohort, relative invasiveness (ρ/D) was significant in the overall patient population and neared significance in the nodular cohort.

**Table 2:** Cox Proportion-Hazards Regression for Survival Data.

(A) Multivariate Regression Analysis				
	Coxph assumption (All patients, N = 245)	p-value (All Patients (N = 245)	Coxph assumption (GBM only, N = 219)	p-value (GBM only, N = 219)
**Global(Age+KPS+…+Steroid)**	0.18	0.0000**	0.27	0.0000**
**Age**	0.01∼	0.0001∼	0.03∼	0.0001∼
**KPS**	0.79	0.0001**	0.83	0.0005**
**XRT Dose**	0.05	0.0000**	0.15	0.0000**
**Concurrent TMZ**	0.31	0.78*	0.18	0.99*
**ρ/D**	0.19	0.04**	0.26	0.12*
**Grade**	0.9	0.0004**	N/A	N/A
**Race**	0.55	0.15*	0.31	0.26*
**EORCriteria**	0.14	0.0000**	0.17	0.0000**
**Gender**	0.83	0.83*	0.97	0.84*
**Dx In 90s**	0.95	0.35*	0.93	0.42*
**Dx In 2000s**	0.55	0.03**	0.45	0.03**
**Pre-op Steroid**	0.69	0.12*	0.92	0.11*
**T1Gd**	0.12	0.81*	0.26	0.83*
**T2**	0.23	0.48*	0.26	0.35*

(B) Univariate Regression Analysis				
	Coxph assumption (All patients, N = 245)	p-value (All Patients (N = 245)	Coxph assumption (GBM only, N = 219)	p-value (GBM only, N = 219)
**Age**	0.002∼	0.0000∼	0.0008∼	0.0000∼
**KPS**	0.0007∼	0.0000∼	0.005∼	0.0000∼
**XRT Dose**	0.04∼	0.01∼	0.59	0.0009**
**Concurrent TMZ**	0.02∼	0.002∼	0.15	0.0000**
**ρ/D**	0.17	0.86*	0.29	0.94*
**Grade**	0.39	0.0000**	N/A	N/A
**Race**	0.02∼	0.03∼	0.008∼	0.18∼
**EORCriteria**	0.0003∼	0.009∼	0.0007∼	0.0002∼
**Gender**	0.59	0.95*	0.4	0.54*
**Dx In 90s**	0.39	0.0000**	0.22	0.0001**
**Dx In 2000s**	0.51	0.0000**	0.32	0.0002**
**Pre-op Steroid**	0.06	0.44*	0.11	0.45*
**T1Gd**	0.52	0	0.44	0.02**
**T2**	0.32	0.01*	0.61	0.02**

Cox Proportion-Hazards Regression Analysis for Survival Data, multivariate (A) and univariate (B) analysis.

* indicates that Cox proportional hazard (Coxph) assumptions were met, and that the given variable does not have a significant effect on survival at the P = 0.05 significance level.

** indicates that Coxph assumptions were met, and that the given variable does have a significant effect on survival at the P = 0.05 level. As expected, variables such as age and KPS have a significant effect on survival.

∼ indicates variables for which the Coxph assumption was not met.

**Figure 4:**
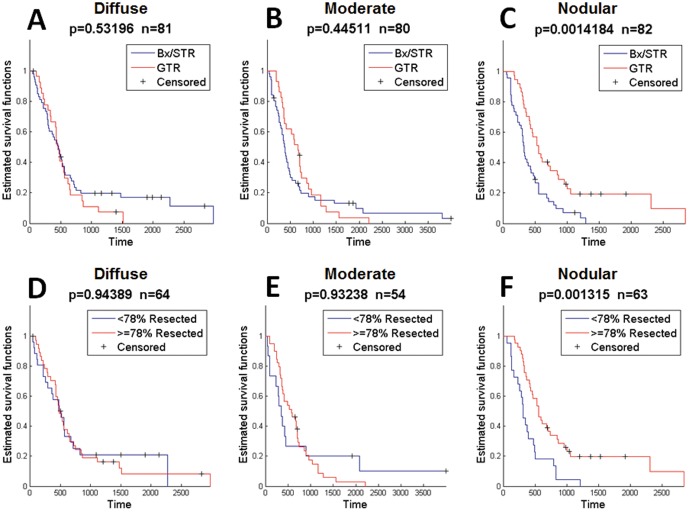
Survival Curves for Highly Diffuse (low ρ/D), Moderately Diffuse (mid ρ/D), and Nodular (high ρ/D) for 243 Contrast-enhancing Gliomas. Comparisons were made between biopsy/subtotal resection (BX/STR) and gross total resection (GTR) in all 243 contrast-enhancing gliomas. A–B) Comparing Bx/STR with GTR in 243 contrast enhancing gliomas at first surgical presentation, there is no significant survival benefit of GTR for highly and moderately diffuse (low and mid ρ/D, p = 0.532, p = 0.445, respectively). C) Patients with the least diffuse and most nodular pattern of growth (high ρ/D) that underwent GTR had a significant survival benefit over patients undergoing Bx or STR (p = 0.00142). Median increase in survival was 227 days (7.5 months) or a 65% improvement over the BX/STR population. When EOR was classified by percent of the T1Gd volume removed, with 76% selected as the cut off, the same selective survival benefit is observed in the nodular cohort (D–F, p = 0.00132). The limits between highly and moderately diffuse, and between moderately diffuse and nodular ρ/D were 0.376 mm^−2^ and 1.30 mm^−2^, respectively.

Analysis of the pre-operative images of all contrasting-enhancing gliomas at initial diagnosis revealed that patients with diffuse and moderate did not receive a significant survival benefit from GTR compared to Bx/STR (p = 0.53, p = 0.45, respectively, Figure 4a–b). In contrast, patients with the least invasive and most nodular of the contrast enhancing gliomas (high ρ/D) survived significantly longer if they received GTR versus Bx/STR (p = 0.001, Figure 4c). This median difference represents a survival advantage of 227 days, a 7.5 month or 65% improvement over the Bx/STR cohort. To be consistent with recent publications suggesting a threshold of removal of 78% of the T1Gd abnormality as being necessary to achieve significant overall survival benefit in a cohort of patients [2], we assessed quantitative measures of EOR based on volumetric quantification pre and post-resection in a subset of our cohort for which such imaging was available (N = 181). Resection was quantified for the subset of patients with sufficient imaging, and resection was categorized as more or less extensive based on a 78% cutoff. This quantification of tumor volume resected displays the same selective survival benefit in the nodular cohort (diffuse: p = 0.94, moderate: p = 0.93, nodular: p = 0.001, Figure 4d–f).

**Figure 5:**
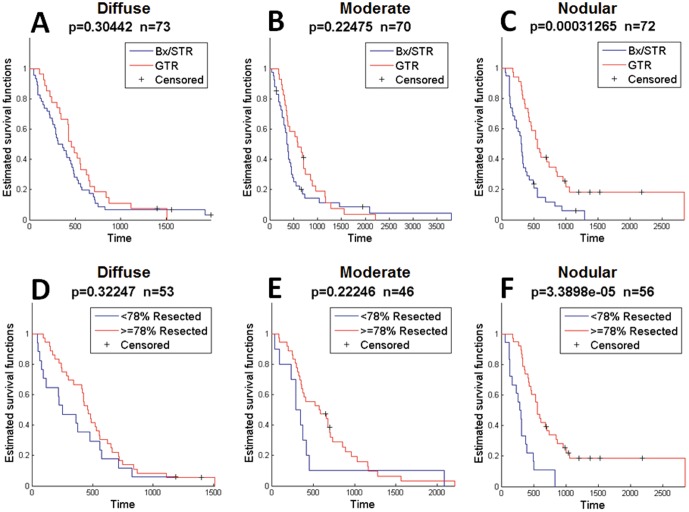
Survival Curves for Highly Diffuse (low ρ/D), Moderately Diffuse (mid ρ/D), and Nodular (high ρ/D) for 215 WHO Grade IV glioblastoma multiforme tumors (GBMs) with sufficient imaging. Comparisons were made between biopsy/subtotal resection (BX/STR) and gross total resection (GTR) in GBMs at first surgical presentation. GTR imparted no significant survival benefit in the highly diffuse (A) and moderately diffuse (B) GBMs. C) GTR imparted a significant survival benefit among patients with nodular GBMs, with a benefit of 239 days (8 months), or a 75% improvement over the Bx/STR group. When EOR was classified by percent of the T1Gd volume removed, with 76% selected as the cut off, the same selective survival benefit is observed in the nodular cohort (D–F, p = 0.0000339). The limits between highly diffuse and moderately diffuse, and between moderately diffuse and nodular ρ/D were 0.439 mm^−2^ and 1.36 mm^−2^, respectively.

Since we restricted our initial analysis to all CE tumors to reflect the reality that the surgeon does not know the grade prior to completing surgery, we wanted to confirm that our results were not skewed by a small number of low-grade gliomas within the population. By limiting the analysis to only the WHO grade IV GBMs (N = 220), we found that, again, GTR imparts no significant survival advantage in patients with more invasive GBMs (Figure 5a, b). In contrast, GTR does impart a significant survival advantage among patients with the least invasive nodular GBMs (p = 0.0003, Figure 5c), providing a median increase in survival time of 239 days (8 months). This 8 month median survival benefit is equivalent to an 80% improvement in median survival time for these patients over the Bx/STR cohort. Similar to the BX/STR vs GTR comparison, we found that only the nodular cohort of GBM patients displayed a significant survival benefit from resection of greater than or equal to 78% of the T1Gd abnormality (p = 0.00003, Figure 5d–f).

Our computational analysis allows us to quantitatively estimate the amount of tumor remaining post-resection including areas peripheral to the imaging abnormality – cf., Figure 1. We are able to generate simulated resections informed by the estimates of cellular density simply with measurements of ρ/D and radius of the visible tumor on T1Gd MRI. Fully acknowledging that surgical planning is a complex clinical process and that each patient may not be a candidate for the extent of resection the model predicts, Figure 3a represents the increase in volume of resection needed over what would be considered a GTR on imaging alone to leave 1% of glioma cells remaining (2 log cell kill) after such an extensive resection, featuring 2 illustrative patients in Figures 3b and 3c. GTR of the T1Gd area in nodular (high ρ/D) lesions removes nearly 99% of the tumor cells, but the additional resection volume would need to be extended significantly for patients with diffuse (low ρ/D) gliomas (Figure 3a). Figure 3b illustrates this point for a representative patient with diffuse disease (low ρ/D). That is, to achieve a cytoreductive effect of resection to remove 99% of the tumor cells diffusely extended in the brain would require a highly unrealistic 237cc of additional tissue to be removed peripheral to the imaging abnormality seen on T1Gd. In contrast, only 25cc of additional tissue would have to be removed in order to achieve the same 99% reduction in the patient with high ρ/D shown in Figure 4c. These illustrative patient cases and the relationship quantified in Figure 3a shows that the most diffuse gliomas require a dramatically increased tumor margin whereas the most nodular gliomas require only a modest increase to attain a minimal residual of 1% of the total glioma cells in the brain.

**Figure 6:**
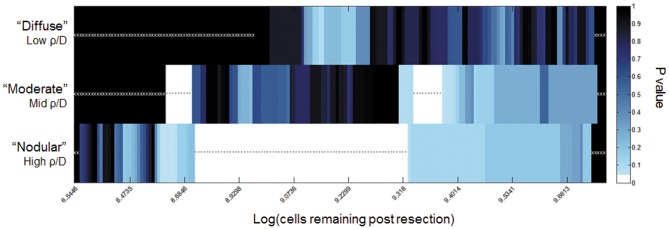
Results of iterative Kaplan-Meier Analysis in each invasiveness cohort. Number of cells remaining was calculated for each patient, based on their ρ/D and measured residual enhancing disease. Each possible threshold was iterated through to separate the patients into large and small residual tumor cell population cohorts. White boxes correspond to thresholds separating patients into groups with significantly different (p<0.05) survival. White stars indicate tests with no p-value, as the threshold did not separate the patients in the given invasiveness cohort into two groups. Black asterisks indicate tests with p<0.05. Black bins with white x's indicate no the threshold did not separate the patients in the given invasiveness cohort into two groups. For example, for the diffuse case (Figure 3, top row), the black bins with white x's represent the fact that even GTR was unable to achieve a remaining cell burden less than the cutoff (up to approximately 10^9^). Further, amongst the most diffuse gliomas, no threshold for a residual cells following resection was found to be significant of outcome represented visually as the lack of a white bar in the top row. Although less dramatic, the moderate cohort was unable to equate a GTR with <10^8.5^ cells remaining represented by the black bars with white x's to the left on middle row of Figure 3. While resection of tumors in the nodular cohort were able to attain residual disease burdens at all levels down to <10^7^ cells.

Combining the model with patient-specific measures of relative invasion, ρ/D, the calculated extent of resection were used to estimate the number of cells remaining post-resection for each patient (N = 181). To determine if there is a threshold of glioma cells remaining following resection that portends better outcomes, iterative Kaplan-Meier analysis was performed using each possible threshold to separate invasiveness cohorts into groups with relatively small and large residual cell populations (Figure 6). The moderate and nodular cohorts (mid and high ρ/D, respectively) show robust thresholds for identifying patients with longer survival (white bins with black asterisks).

## Discussion

Our results show that for patients with less diffuse, nodular tumors (high ρ/D), GTR imparts a significant survival benefit; whereas GTR does not afford any significant survival benefit in patients with more diffuse tumors. Though other studies have seen a modest population-scale survival benefit with increasingly extensive resections of the T1Gd abnormality [1], [2], our results suggest that the difference seen in the survival curves when considering the entire population of GBMs may have been driven by the subset of GBM patients with less diffuse disease. Therefore, the model-based estimate of tumor invasiveness that we used for our analysis (ρ/D) could potentially act as a metric for identifying glioma patients that are more likely to benefit from GTR. Although the approach described here relies on detailed, quantitative, computational representations of the pre-operative tumor, our method basically synthesizes and quantifies the neurosurgical intuition as to which patients will most likely benefit from GTR. In addition to basic patient data such as age and KPS and symptomatic considerations such as intracranial pressure reduction, the individual tumor growth characteristics applied here can also inform the physician's perspective on each patient's illness. Understanding the behavior of the disease is necessary for understanding how the tumor will respond to treatment. This motivates a future prospective validation study in which the optimal ρ/D cut-off and simulations of tumor growth after varying extents of resection will improve our ability to determine the likely benefit gained from surgical intervention.

Further study is needed to understand the degree to which gross total resection or more extensive resection should be advocated in patients for which functional deficits are likely to result from surgery, as previous studies have shown that such deficits poorly impact survival [30]. Although recent studies have suggested that more aggressive resections are most beneficial, these GTRs also carry a higher risk of immediate post-operative deterioration [31]. Understanding the extent of cytoreduction achieved by aggressive surgery for each patient will help surgeons evaluate the potential benefit in taking the known risk associated with this treatment.

As technological innovations continue to propel surgeons' ability to achieve greater EOR, it will become increasingly important to identify the subset of glioma patients who will truly benefit from gross total resection [7]. Since the degree of benefit appears to be so profound in patients with high ρ/D (75—80% improvement over the STR/Biopsy cohort), these results suggest that a subtle selection bias towards these relatively less invasive gliomas in any uncontrolled or small study could lead to over estimation of robust median survivals with no true clinical benefit. Incorporation of work by the RANO group to better define and consider postoperative changes on MRI could further improve EOR classification [32]. Given the advent of intraoperative imaging as a means of confirming resection of imageable disease, these methods could develop as an adjunct to such approaches that incorporate knowledge of the diffuse extent of tumor invasion peripheral to the imaging abnormality. Our results suggest that a prescribed subset of patients may benefit from surgical resection that extends beyond the margins defined by T1Gd MRI. We envision that a new neurosurgical planning tool that combines our modeling approach with functional mapping could better define the surgical margins that maximize cytoreduction while preserving neurological function. This could be combined with recent tools we have developed to use patient-specific simulations of tumor growth to generate metrics of treatment response found that are prognostically significant [28], [33] to begin a patient-centered suite of tools for treatment decision support.

This study also suggests future analyses to determine if there are other biological features of less diffuse (high ρ/D) patients that make them more responsive to GTR. We have previously shown that these nodular tumors are more likely to be hypoxic [21] and may have worse prognosis overall [20] but paradoxically receive a larger benefit from aggressive treatment [18]. This may be due to a combination of factors, such as the observation that nodular (high ρ/D) patients tend to have larger net proliferation rates (ρ) and thus the time course to significant tumor recurrence is decreased for such aggressive tumors.

## Conclusions

Using pre-operative MRI to quantify the invasion profile, patients with low relative invasiveness are most likely to benefit from GTR with a striking 80% improvement in survival over their STR/Bx controls. This provides a practical therapeutic strategy for neurosurgeons in the context of invasive gliomas, and a method for cohorting patients in clinical trial design to identifying the cohort of patients benefiting strongly from GTR. By characterizing the disease itself in a patient-specific manner, we are able to inform a long-standing debate as to the role of resection in the treatment of diffuse gliomas.

## Supporting Information

Table S1 **Cox Proportion-Hazards Regression for Cohorted Survival Data.**
(DOC)
