# Supplementary material for: Patient-Specific Metrics of Invasiveness Reveal Significant Prognostic Benefit of Resection in a Predictable Subset of Gliomas
Source: PLoS One. 2014 Oct 28;9(10):e99057. doi: 10.1371/journal.pone.0099057 (PMC4211670; doi:10.1371/journal.pone.0099057)
Supplement: Table S1 — Cox Proportion-Hazards Regression for Cohorted Survival Data. (DOC) [file pone.0099057.s001.doc]

| **Table S1 - Cox Proportion-Hazards Regression for Cohorted Survival Data** | | | | | | | |
| --- | --- | --- | --- | --- | --- | --- | --- |
|  | | **Low p/D group** | | **Mid p/D group** | | **High p/D group** | |
|  |  | **coxph assumption** | **p-value** | **coxph assumption** | **p-value** | **coxph assumption** | **p-value** |
| **Multivariate regression analysis** | **Global(Age + KPS + ... + Steroid)** | 0.2301 | 0.0000 | 0.7774 | 0.0000 | 0.0683 | 0.0000 |
|  | **Age** | 0.1470 | 0.0029 | 0.2999 | 0.0021 | 0.0418 | 0.1762 |
|  | **KPS** | 0.9659 | 0.0088 | 0.5376 | 0.0263 | 0.2356 | 0.0436 |
|  | **XRT Dose** | 0.0742 | 0.1826 | 0.2989 | 0.0046 | 0.1175 | 0.3388 |
|  | **Concurrent TMZ** | 0.4119 | 0.9691 | 0.9095 | 0.7516 | 0.3533 | 0.8881 |
|  | **ρ/D** | 0.0406 | 0.2778 | 0.4133 | 0.8473 | 0.1704 | 0.0555 |
|  | **Grade** | 0.4699 | 0.1700 | 0.2673 | 0.0005 | 0.3198 | 0.4986 |
|  | **Race** | 0.3442 | 0.0435 | 0.5785 | 0.0732 | 0.1115 | 0.3580 |
|  | **EORCriteria** | 0.0736 | 0.3900 | 0.0988 | 0.0000 | 0.3574 | 0.5399 |
|  | **Gender** | 0.5602 | 0.6771 | 0.7924 | 0.1301 | 0.1490 | 0.4848 |
|  | **Dx In 90s** | 0.2773 | 0.3002 | 0.9820 | 0.7441 | 0.0143 | 0.2924 |
|  | **Dx In 2000s** | 0.6890 | 0.7245 | 0.7223 | 0.1117 | 0.0218 | 0.1856 |
|  | **Pre-op Steroid** | 0.2737 | 0.8212 | 0.5500 | 0.4963 | 0.2379 | 0.0025 |
|  | **T1Gd** | 0.4721 | 0.9333 | 0.2593 | 0.5989 | 0.4266 | 0.6788 |
|  | **T2** | 0.4316 | 0.7707 | 0.2655 | 0.5512 | 0.5460 | 0.5825 |
| **Univariate regression analysis** | **Age** | 0.0215 | 0.0000 | 0.1260 | 0.0068 | 0.1280 | 0.0014 |
|  | **KPS** | 0.1210 | 0.0000 | 0.0224 | 0.2490 | 0.0810 | 0.0000 |
|  | **XRT Dose** | 0.0238 | 0.4840 | 0.6310 | 0.0056 | 0.4620 | 0.9360 |
|  | **Concurrent TMZ** | 0.0495 | 0.1170 | 0.4200 | 0.0632 | 0.2120 | 0.0398 |
|  | **ρ/D** | 0.5330 | 0.1080 | 0.5590 | 0.6570 | 0.1590 | 0.8570 |
|  | **Grade** | 0.4860 | 0.0008 | 0.4670 | 0.0631 | 0.4680 | 0.1330 |
|  | **Race** | 0.6630 | 0.5140 | 0.8580 | 0.4650 | 0.0006 | 0.0212 |
|  | **EORCriteria** | 0.0300 | 0.7300 | 0.0150 | 0.1810 | 0.1960 | 0.0028 |
|  | **Gender** | 0.5130 | 0.4390 | 0.7650 | 0.2630 | 0.6340 | 0.4800 |
|  | **Dx In 90s** | 0.6950 | 0.0013 | 0.7920 | 0.1290 | 0.4580 | 0.0000 |
|  | **Dx In 2000s** | 0.8940 | 0.0018 | 0.6680 | 0.1070 | 0.1760 | 0.0002 |
|  | **Pre-op Steroid** | 0.4710 | 0.1040 | 0.5280 | 0.4450 | 0.0386 | 0.3190 |
|  | **T1Gd** | 0.8460 | 0.0308 | 0.6690 | 0.2120 | 0.7440 | 0.0000 |
|  | **T2** | 0.7080 | 0.4080 | 0.6550 | 0.2030 | 0.8510 | 0.0004 |
| P value greater than 0.05 | | P value less than 0.05 | | | Coxph Assumption not met | | |

**Table S1 Cox Proportion-Hazards Regression Analysis for Cohorted Survival Data, multivariate and univariate analysis.** Green indicates that Cox proportional hazard (Coxph) assumptions were met, and that the given variable does not have a significant effect on survival at the P=0.05 significance level. Orange indicates that Coxph assumptions were met, and that the given variable does have a significant effect on survival at the P=0.05 level. As expected, variables such as age and KPS have a significant effect on survival. Grey indicates variables for which the Coxph assumption was not met.
